# Personality Traits, Loneliness, and Affect Among Boxers

**DOI:** 10.3389/fpsyg.2021.609153

**Published:** 2021-02-18

**Authors:** Xin Chen, Nan Qiu, Chao Chen, Liang Zhai

**Affiliations:** ^1^Department of Sport and Health Sciences, Technical University of Munich, Munich, Germany; ^2^General and Experimental Psychology, Department of Psychology, LMU Munich, Munich, Germany; ^3^School of Physical Education and Sport Training, Shanghai University of Sport, Shanghai, China; ^4^Shanghai Key Lab of Human Performance, Shanghai University of Sport, Shanghai, China; ^5^College of Physical Education, Sichuan Agricultural University, Yaan, China; ^6^Institute of Sport Science, Southwest University, Chongqing, China

**Keywords:** big five—personality, loneliness, positive affect, negative affect, mediating effect, boxers

## Abstract

This study aimed to test the association between personality traits and affect among boxers and to figure out whether loneliness mediated this relationship. This study used The Big Five Personality Traits Scale, The UCLA Loneliness Scale, and The Positive and Negative Affect Scale (PANAS) which were administered to *N* = 231 boxers (age: M = 20.28; *SD* = 2.60), of which 62% were male (*n* = 144) and 38% were female (*n* = 87). The results showed that (1) conscientiousness, extraversion, and agreeableness were negatively related with negative affect, neuroticism was positively associated with negative affect, and openness showed no relationship with negative affect; (2) conscientiousness, extraversion, and agreeableness were all positively correlated with positive affect, neuroticism was negatively associated with positive affect, and openness showed no relationship with positive affect; (3) conscientiousness, extraversion, openness, and agreeableness were negatively related with loneliness and neuroticism was positively associated with loneliness; and (4) loneliness was positively associated with negative affect; loneliness was negatively associated with positive affect; and loneliness had mediating effect on the relationship between the personality traits and affect. Furthermore, these findings suggest that loneliness might be one mechanism explaining how boxer’s personality traits influence their athletic performance. Therefore, in the future, we should start by helping them reduce their loneliness to reduce their negative affect and improve their positive affect and also, in some degree, to enhance boxers’ athletic ability and mental quality health.

## Introduction

Affect is a research focus in the field of psychology, especially concerning how negative affect can influence many aspects of an individual’s life and even accompany the individual’s life, resulting in serious consequences (Ollendick and King, 1994). Mainstream affect theory holds that human affect contains two reciprocal dimensions: positive affect and negative affect. Positive affect refers to all passionate, sober, and lively emotional experiences; negative affect refers to those emotional experiences that are related to pain, numbness, and silence (Ebesutani et al., 2012). The two-factor model is studied from a dimensional perspective, and it is also concluded that affects are divided into positive and negative affect (Zevon and Tellegen, 1982; Tuccitto et al., 2010). The stability and adaptability of this model in different cultural studies have been confirmed (Thompson, 2007), and several studies have revealed that individuals’ positive and negative affects can affect their mental health, life satisfaction, and well-being (Diehl et al., 2011; Kong et al., 2019; Quinn et al., 2019). In addition, some scholars have found that positive and negative affects can predict individual depression, anxiety, and suicidal tendencies (Denollet and De, 2006; Uhl et al., 2019). Previous studies have found that positive and negative affects influence athletes’ coping during burnout and motivation (Gaudreau et al., 2002; Lemyre et al., 2006; Gustafsson et al., 2013). Thus, affect research is important across research fields. Sports psychology researchers advocate attention and research on special groups, including boxers (Schinke, 2007; Simpson and Wrisberg, 2013). Studies have shown that boxers are suffering from psychological distress and loneliness, leading some to terminate their elite careers at a young age (Middleton et al., 2018). Therefore, investigating the influential factors and mechanisms of boxers’ positive and negative affects are particularly important for preventing and reducing such negative affect and promote their healthy development.

Eysenck or Gray/Newman are models for understanding personality-affect relationships (Rusting and Larsen, 1997). Personality is one of the most robust predictors of positive affect and negative affect (DeNeve and Cooper, 1998; Diener and Lucas, 1999). One highly influential model describing personality structure is the Big Five Personality model, which includes extraversion, agreeableness, conscientiousness, neuroticism, and openness (John et al., 1991). Many studies have found that neuroticism is significantly positively correlated with negative affect but not correlated with positive affect (Meyer and Shack, 1989; Rusting and Larsen, 1995). A longitudinal study showed that neuroticism and extraversion can significantly predict positive and negative affects (Finch et al., 2012; Wang et al., 2009). Meta-analytic investigations show that extraversion and positive affect are positively correlated, and neuroticism significantly predicts negative affect (Steel et al., 2008). Some studies have shown that agreeableness is positively correlated with positive affect, and agreeableness can negatively predict negative affect (Panaccio and Vandenberghe, 2012). Previous studies explicated that conscientiousness positively predicts positive affect and negatively predicts negative affect; but openness is not related to positive or negative affect (Berry et al., 2000). This study investigated the relationship between boxers’ personality traits and affect to further clarify the value of boxers’ personality traits and to provide theoretical guidance for the prevention and intervention of boxers’ negative affect.

Many studies have shown that personality traits and loneliness are closely related in various populations (Abdellaoui et al., 2019; Buecker et al., 2020). Loneliness does not refer to a state of being alone but to a subjective emotional experience when one perceives that their social relationship is not what they expected and they cannot obtain satisfaction from social interactions (Heinrich and Gullone, 2000). Previous studies have elucidated that individuals’ extraversion is significantly negatively correlated with loneliness (Hensley et al., 2012; Vanhalst et al., 2012; Teppers et al., 2013). Extroverts have good interpersonal relationships and prefer to participate in social life; therefore, their loneliness levels may be relatively low (Mervielde and De, 2000; Van et al., 2010; Nikitin and Freund, 2015). Studies have confirmed that agreeableness negatively predicts loneliness (Stokes, 1985; Saklofske and Yackulic, 1989). Regarding the relationship between conscientiousness and loneliness, some studies have found positive associations, others have found negative associations, and still others have found no significant association (Vanhalst et al., 2012; Teppers et al., 2013; Mund and Neyer, 2018). The research has consistently found that neuroticism and loneliness are significantly negatively correlated (Hensley et al., 2012; Vanhalst et al., 2012). Regarding the relationship between openness and loneliness, some previous studies found negative associations while others found no significant association (Saklofske and Yackulic, 1989; Vanhalst et al., 2012; Teppers et al., 2013). Based on the above research, exploring the relationship between personality traits and boxers’ loneliness has become particularly important to further understand the psychological characteristics of boxers.

The close relationship between loneliness and affect has been confirmed among different groups (Ernst and Cacioppo, 1999). A longitudinal study found that individual loneliness significantly negatively predicts positive affect and significantly positively predicts negative affect (Joiner, 1997). There is much evidence of a negative relationship between positive affect and loneliness (Aanes et al., 2009; Van et al., 2014). Scholars have found that the loneliness of adolescents has a strong negative predictive effect on positive affect (Einav et al., 2018). Some studies have found that positive affect decreases as individual loneliness levels increase (Ditcheva et al., 2018), and subsequent studies have confirmed, through longitudinal research, that loneliness can negatively predict positive affect at different ages (Bennardi et al., 2019). On the other hand, studies have revealed that loneliness and negative affect are significantly positively correlated (Lee et al., 2017). Recent experimental studies found that loneliness can significantly predict individual negative affect, providing a new explanation for the relationship between the two (Wong et al., 2019). The relationship between personality traits, loneliness, and affect has thus received academic attention, but how loneliness affects the mechanism of personality traits and affect still needs further discussion. Therefore, this study will focus on the relationship between boxers’ loneliness and affect and whether loneliness has a significant mediating role between personality traits and affect.

**FIGURE 1:**
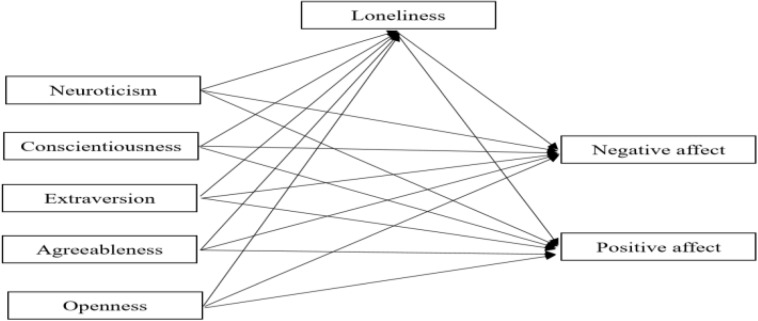
The proposed mediation model.

Based on previous studies, we propose the following hypotheses: (H1) conscientiousness, extraversion, and agreeableness were negatively related with negative affect, neuroticism was positively associated with negative affect, and openness showed no relationship with negative affect; conscientiousness, extraversion, and agreeableness were all positively correlated with positive affect, neuroticism was negatively associated with positive affect, and openness showed no relationship with positive affect; and (H2) conscientiousness, extraversion, openness, and agreeableness were negatively related with loneliness and neuroticism was positively associated with loneliness; and (H3) loneliness was positively associated negative affect, loneliness was negatively associated with positive affect; and loneliness mediates the relationship between personality traits (extraversion, agreeableness, conscientiousness, openness, and neuroticism) and positive and negative affects. Figure 1 illustrates the conceptual model applied.

## Materials and Methods

### Participants

Two hundred thirty-one boxers participated in this study, of which 62% were males (*n* = 144) and 38% were females (*n* = 87). The average age was M age = 20.28, SD age = 2.60, and average prior training period was *M* = 6.07 years (*SD* = 2.90) competing in national- and regional-level competitions, indifferent types of individual, and team/club.

### Measures

#### The Big Five Personality Inventory

The Big Five Personality Inventory (BFPI) (John et al., 1991) is a 44-item measure that consists of the following five personality factors: extraversion (e.g., “Is full of energy”), agreeableness (e.g., “Is helpful and unselfish with others”), conscientiousness (e.g., “Perseveres until the task is finished”), neuroticism (e.g., “Is emotionally stable, not easily upset”), and openness (e.g., “Is original, comes up with new ideas”). The self-report items are rated on a 5-point Likert scale ranging from “strongly disagree (1)” to “strongly agree (5).” The internal consistencies of the inventory’s five scales were α = 0.67 (Neuroticism), α = 0.68 (Agreeableness), α = 0.67 (Conscientiousness), α = 0.72 (Extraversion), and α = 0. 65 (Openness). The factor loadings of the items ranged from *a* = 0.44 to *a* = 0.75. The BFPI has been shown to have satisfactory reliability and validity (Liu and Zheng, 2019).

### The UCLA Loneliness Scale

Loneliness was assessed using the UCLA Loneliness Scale (Version 3) (Russell, 1996), which has been found to be both reliable and valid among China samples (Tan et al., 2016). The UCLA Loneliness Scale comprises 20 items such as “Do you feel intimate with anyone?” and “Do you feel that no one understands you?” Participants rated each item on a 4-point scale, ranging from 1 (never) to 4 (always). Higher scores represent higher levels of loneliness. In the current study, the factor loadings of the items ranged from *a* = 0.32 to *a* = 0.72, and the internal consistencies of this scale were α = 0.87.

### The PANAS Scale

The PANAS Scale is composed of a 10-item subscale assessing positive affect (PA; e.g., attentive, strong, and enthusiastic) and a 10-item subscale measuring negative affect (NA; e.g., guilty, scared, and hostile). Athletes responded to the stem “Indicate the extent to which you generally experience the items listed below during training/practice in the past week.” Responses were rated on a 5-point Likert scale (Watson et al., 1988) ranging from (1) not at all to not at all to not at all (5) extremely. Subscale items were averaged to form weekly PA and NA scores. Previously validated in sport settings (Crocker, 1997), the PANAS is pertinent as it offers an orthogonal perspective to the study of negative and positive affective states in athletes. In the current study, the factor loadings of the items ranged from *a* = 0.41 to *a* = 0.76, the internal consistencies of this scale were PA α = 0.80 and NA α = 0.82.

### Procedures

This study complies with ethics procedures, and the whole procedure was conducted online. The questionnaire survey was conducted after the participants’ consent, and it took 20 min in total. This study emphasizes anonymous and voluntary participation in the survey. We set the participants to click “Yes, I agree to participate in this survey” to prove that they are willing to participate before the questionnaire starts. If the participants click “No, I don’t want to participate in this survey,” they prove that they are not willing to participate in the survey, and the survey ends immediately.

### Data Analysis

We first presented descriptive statistics and bivariate correlations for variables of interest using SPSS 22.0. Then, we examined whether loneliness mediates the relationship between personality traits and affect. The analysis of moderated mediation model was performed using Hayes’ PROCESS macro (Hayes, 2017), which has been used to examine whether the magnitude of a mediation effect is conditional on the value of a moderator. The bootstrapping method produced 95% bias-corrected confidence intervals (CIs) of effects from 5,000 resamples of the data (sample size1/4 of 200). CIs that do not contain zero indicate significant effects.

## Results

### Correlational Analysis

Means, standard deviations, and correlations among the study variables are presented in Table 1. Specifically, correlation results demonstrated that conscientiousness, extraversion, openness, and agreeableness were all positively correlated with positive affect and neuroticism and negatively correlated with positive affect; conscientiousness, extraversion, and agreeableness were all negatively correlated with negative affect and neuroticism and positively correlated with negative affect; and openness is not significantly related to negative affect. Besides, conscientiousness, extraversion, openness, and agreeableness were all negatively correlated with loneliness and neuroticism was positively correlated with loneliness. Furthermore, positive affect was negatively correlated with negative affect and loneliness and positively correlated with negative affect and loneliness and negatively correlated with positive affect. As such, H1, H2, and H3 were confirmed.

**TABLE 1:** Means, standard deviations, and correlation coefficients of personality traits, loneliness, positive affect, and negative affect.

	**M**	***SD***	**1**	**2**	**3**	**4**	**5**	**6**	**7**	**8**
Neuroticism	2.75	0.59	–							
Extraversion	3.45	0.53	−0.52*	–						
Agreeableness	3.77	0.56	−0.42*	0.52*	–					
Conscientiousness	3.42	0.53	−0.52*	0.46*	0.47*	–				
Openness	3.47	0.54	−0.38*	0.56*	0.53*	0.42*	–			
Loneliness	2.01	0.45	0.31*	−0.35*	−0.34*	−0.21*	−0.18*	–		
Positive affect	3.15	0.67	−0.41*	0.39*	0.33*	0.35*	0.39*	−0.47*	–	
Negative affect	2.06	0.65	0.37*	−0.23*	−0.21*	−0.21*	–0.12	0.43*	−0.32*	–

*Indicates p < 0.05.

*Statistically significant.

### Testing for Mediation Effect

Hypothesis 3 stated that loneliness mediates the relationship between personality traits and affect. The PROCESS macro (Model 4) developed by Hayes (2017) was used to test this mediation effect. The following path results partially supported our hypotheses 3.

The path results showed 9 significant indirect mediating effects (see Table 2): (1) loneliness significantly mediated the relationship between neuroticism and positive affect (β = −0.13, 95% CI = [−0.22, −0.06]); (2) loneliness significantly mediated the relationship between extraversion and positive affect (β = 0.17, 95% CI = [0.09, 0.25]); (3) loneliness significantly mediated the relationship between agreeableness and positive affect (β = 0.17, 95% CI = [0.10, 0.25]); (4) loneliness also significantly mediated the relationship between conscientiousness and positive affect (β = 0.11, 95% CI = [0.03, 0.20]); (5) loneliness also significantly mediated the relationship between openness and positive affect (β = 0.09, 95% CI = [0.03, 0.18]); (6) loneliness significantly mediated the relationship between neuroticism and negative affect (β = 0.12, 95% CI = [0.07, 0.19]); (7) loneliness significantly mediated the relationship between extraversion and negative affects (β = −0.17, 95% CI = [−0.28, −0.08]); (8) loneliness significantly mediated the relationship between agreeableness and negative affect (β = −0.16, 95% CI = [−0.26, −0.09]); and (9) loneliness significantly mediated the relationship between conscientiousness and negative affect (β = −0.10, 95% CI = [−0.20, −0.03]).

**TABLE 2:** Indirect mediating results.

	**β**	**SE**	**95% CI**
Neuroticism → loneliness → positive affect	–0.13	0.04	−0.22, −0.06
Extraversion → loneliness → positive affect	0.17	0.04	0.09, 0.25
Agreeableness → loneliness → positive affect	0.17	0.04	0.10, 0.25
Conscientiousness → loneliness → positive affect	0.11	0.04	0.03, 0.20
Openness → loneliness → positive affect	0.09	0.04	0.03, 0.18
Neuroticism → loneliness → negative affect	0.12	0.03	0.07, 0.19
Extraversion → loneliness → negative affect	–0.17	0.05	−0.28, −0.08
Agreeableness → loneliness → negative affect	–0.16	0.04	−0.26, −0.09
Conscientiousness → loneliness → negative affect	–0.10	0.04	−0.20, −0.03

The results demonstrated that boxer loneliness significantly mediates the relationship between personality traits and positive affect, and loneliness significantly mediates the relationship between personality traits and negative affect.

## Discussion

From the perspective of Eysenck or Gray/Newman models, this study explored the relationship and internal mechanism of personality and affect. The correlation results demonstrated that conscientiousness, extraversion, openness, and agreeableness were all positively correlated with positive affect and neuroticism and negatively correlated with positive affect; conscientiousness, extraversion, and agreeableness were all negatively correlated with negative affect and neuroticism and positively correlated with negative affect; and openness is not significantly related to negative affect. This is consistent with previous results (Rusting and Larsen, 1995; Steel et al., 2008; Finch et al., 2012; Panaccio and Vandenberghe, 2012). This study further confirmed that boxer’s personality can significantly predict affect, providing a new perspective for the research in the field of sports science. Extraversion people like to be in touch with people, are full of energy, and often feel positive emotions (Friedman and Schustack, 2016); previous studies on extraversion showed a significant positive correlation with positive affect and a negative correlation with negative affect (Wang et al., 2009; Finch et al., 2012). Individuals with high neuroticism tend to have psychological pressure, unrealistic thoughts, excessive demands, and impulses and are more likely to experience negative emotions such as anger, anxiety, depression, etc. (Jeronimus et al., 2014). Through longitudinal research and meta-analytic research, scholars have found that neuroticism significantly predicts positive affect negatively and positively predicts negative affect (Steel et al., 2008; Wang et al., 2009). People with high agreeableness are optimistic about human nature and believe that human nature is good (Rothmann and Coetzer, 2003); some studies also prove that agreeableness is positively correlated with positive affect and negatively correlated with negative affect (Panaccio and Vandenberghe, 2012). Regarding the relationship between conscientiousness, openness, and affect, scholars have found that conscientiousness positively predicts positive affect and negatively predicts negative affect; however, openness is not related to positive and negative affects (Berry et al., 2000). Therefore, this study explores the relationship between the boxer’s personality traits and affect and expands the related research in this field, which has a positive driving role and important guiding significance for the boxer’s physical and mental health development and negative affect intervention.

To test the hypothesis of this study, mediating effect test was conducted. The mediating effect test shows that loneliness has a mediating role between personality traits and affect.

First, personality factor is a more essential psychological and behavioral system, which internally restricts and determines the unique tendencies and characteristics of individual behavioral activities (Allport, 1937), and loneliness is the difference between the individual’s perception of the actual social status and the expected social status, or the inability to establish an emotional bond with important others, and experience negative emotions (Bauminger and Kasari, 2000). Most studies have found that personality traits are closely related to individual loneliness (Abdellaoui et al., 2019; Schermer and Martin, 2019; Schutter et al., 2020). This study shows that boxers’ conscientiousness, extraversion, openness, and agreeableness were all negatively correlated with loneliness and their neuroticism was positively correlated with loneliness. These findings support hypothesis 2. Moreover, individuals with high extraversion prefer social activities in life and work, which may lower this group of people’s loneliness (Horstmann et al., 2020); conversely, individuals with high neuroticism are repulsive to social activities, which may lead to high loneliness (Denissen and Penke, 2008). Previous studies have shown that conscientiousness, openness, and agreeableness significantly predict loneliness in the negative direction (Vanhalst et al., 2012; Teppers et al., 2013; Mund and Neyer, 2018), which is also confirmed in boxers.

Second, loneliness is also one of the effective predictors for predicting individual affects (Ernst and Cacioppo, 1999). The relevant analysis of this study shows that loneliness was positively correlated with negative affect and negatively correlated with positive affect. These findings support hypothesis 3. Scholars have found that loneliness of individuals can significantly predict positive affect negatively in both horizontal and vertical studies, and individuals with high loneliness have low positive affect in different groups (Ditcheva et al., 2018; Einav et al., 2018; Bennardi et al., 2019). In addition, individual’s loneliness and negative affect having a significant positive correlation has been confirmed (Lee et al., 2017; Wong et al., 2019). In order to further investigate whether loneliness plays a mediating role in the relationship between boxers’ personality traits and affect, this study used the bootstrapping method to test the possible mediating effects of loneliness. The results confirm that loneliness plays a significant mediating role in the relationship between boxers’ personality traits and affect. These results support research hypotheses 3. Boxers’ personality traits influence affect, at least indirectly through loneliness.

## Conclusion

First, there are significant correlations of personality traits with affect. Second, there are significant correlations of personality traits with loneliness; loneliness is negatively correlated with positive affect, and loneliness is positively correlated with negative affect. Finally, boxers’ loneliness mediates personality traits and affect, indicating that personality traits predict positive affect/negative affect not only directly but also indirectly through loneliness. In summary, the results of this study are not only applicable to general boxers. It would also be promoted among elite athletes to prevent loneliness and negative affect during high-pressure training or elite athletes’ competitions.

## Data Availability Statement

The raw data supporting the conclusions of this article will be made available by the authors, without undue reservation.

## Ethics Statement

The studies involving human participants were reviewed and approved by the Southwest University’s Human Research Ethics Committee. Prior to initiation of the study, all subjects gave written informed consent in accordance with the Declaration of Helsinki.

## Author Contributions

XC, NQ, CC, and LZ conceived the study, interpreted the data, drafted and revised the work, approved the final version of the manuscript to be published, and agreed to be accountable for all aspects of the work.

## Conflict of Interest

The authors declare that the research was conducted in the absence of any commercial or financial relationships that could be construed as a potential conflict of interest.
